# Semantic Classical Conditioning and Brain-Computer Interface Control: Encoding of Affirmative and Negative Thinking

**DOI:** 10.3389/fnins.2013.00023

**Published:** 2013-03-07

**Authors:** Carolin A. Ruf, Daniele De Massari, Adrian Furdea, Tamara Matuz, Chiara Fioravanti, Linda van der Heiden, Sebastian Halder, Niels Birbaumer

**Affiliations:** ^1^Institute of Medical Psychology and Behavioral Neurobiology, University of TübingenTübingen, Germany; ^2^Graduate Training Centre of Neuroscience, International Max Planck Research SchoolTübingen, Germany; ^3^IRCCS Fondazione Ospedale San CamilloVenezia, Italy; ^4^Wilhelm-Schickard Institute for Computer Engineering, University of TübingenTübingen, Germany; ^5^Department of Cognitive Psychology, University of Finance and ManagementWarsaw, Poland; ^6^Institute of Psychology, University of WürzburgWürzburg, Germany

**Keywords:** brain-computer interface, classical conditioning, EEG, auditory, semantic conditioning, brain communication

## Abstract

The aim of the study was to investigate conditioned electroencephalography (EEG) responses to factually correct and incorrect statements in order to enable binary communication by means of a brain-computer interface (BCI). In two experiments with healthy participants true and false statements (serving as conditioned stimuli, CSs) were paired with two different tones which served as unconditioned stimuli (USs). The features of the USs were varied and tested for their effectiveness to elicit differentiable conditioned reactions (CRs). After acquisition of the CRs, these CRs to true and false statements were classified offline using a radial basis function kernel support vector machine. A mean single-trial classification accuracy of 50.5% was achieved for differentiating conditioned “yes” versus “no” thinking and mean accuracies of 65.4% for classification of “yes” and 68.8% for “no” thinking (both relative to baseline) were found using the best US. Analysis of the area under the curve of the conditioned EEG responses revealed significant differences between conditioned “yes” and “no” answers. Even though improvements are necessary, these first results indicate that the semantic conditioning paradigm could be a useful basis for further research regarding BCI communication in patients in the complete locked-in state.

## Introduction

One of the purposes of brain-computer interfaces (BCIs) is to enable muscle-independent communication for individuals who are not able to communicate by any other means due to severe paralysis. Several neurological diseases such as amyotrophic lateral sclerosis (ALS), brain stem stroke, or high spinal cord injury may lead to such severe or complete motor paralysis. BCI devices could be controlled through brain signals recorded non-invasively using electroencephalography (EEG) or magnetoencephalography (MEG) to measure brain activity as well as functional magnetic resonance imaging (fMRI) or functional near infrared spectroscopy (fNIRS) to measure blood oxygenated level dependent (BOLD) brain metabolism (Caria et al., 2007; Naito et al., 2007; Sitaram et al., 2007, 2009; Wriessnegger et al., 2008; Battapady et al., 2009; Wang et al., 2010). Likewise, invasive BCIs record brain activity after the implementation of microelectrode arrays or electrocorticography (ECoG) electrodes (Hochberg et al., 2006; Murguialday et al., 2011).

The BCI systems currently being operated and investigated can be assigned to two categories: (i) BCI systems that rely on evoked brain responses (e.g., P300, steady-state evoked potentials) for item selection and require sustained focused attention without extensive previous training (Farwell and Donchin, 1988; Middendorf et al., 2000; Cheng et al., 2002; Nijboer et al., 2008a), and (ii) BCI systems that allow communication and environmental control through voluntary self-regulation of specific brain signals (e.g., slow cortical potentials, sensorimotor rhythm; Kübler et al., 1999; Pfurtscheller et al., 2000; Blankertz et al., 2010). The voluntary learning of self-regulation of specific brain signals for BCI control is achieved by means of operant training. This form of learning, through feedback and reward relies on visual or auditory signals reflecting the brain activity and positive or negative reinforcement.

During the last three decades BCI-related studies have reported successful use of non-invasive EEG-based BCIs among groups of patients with various disabilities (Birbaumer et al., 1999; Kübler et al., 2005; Müller-Putz et al., 2005; Sellers and Donchin, 2006; Vaughan et al., 2006; Buch et al., 2008; Nijboer et al., 2008a; Silvoni et al., 2009). Patients with locked-in syndrome (LIS) are severely paralyzed but have residual voluntary control over particular muscles (e.g., eye muscles, face muscles, fingers). In the complete locked-in state (CLIS), patients lose all communication channels with their environment. Previous attempts to restore or maintain communication with different BCIs were successful in LIS patients (Birbaumer et al., 1999; Kübler et al., 2005, 2009; Nijboer et al., 2008a), but failed in patients in CLIS (Kübler and Birbaumer, 2008). Although patients in CLIS have the most dire need for a BCI, there have been few attempts to investigate the reasons for their failure to achieve BCI control. It has been hypothesized that periods of complete paralysis lead to extinction of goal-directed thinking and voluntary intentions as a consequence of lost reinforcement contingencies between behavior and its feedback. In CLIS patients, thoughts or intentions (to move a limb) are not followed by their anticipated consequences (the limb is not moving as intended) and, over a longer period of time, would then extinguish goal-directed thinking (Birbaumer, 2006; Kübler and Birbaumer, 2008).

The hypothesis of extinction of reward-based learning in paralyzed organisms is supported by findings of experiments on curarized rats (Dworkin and Miller, 1986). These studies have demonstrated the inability of operant (voluntary) learning to control visceral functions under complete paralysis. As the life-sustaining bodily functions of curarized rats were kept constant in these experiments, the homeostatic effect of the reward (rewarding brain stimulation or avoidance from shock) on body functions and, as a consequence, on learning, was absent. Importantly, Dworkin showed that despite the absence of instrumental learning, classical conditioning of curarized rats was possible. After pairing tones with aversive stimuli they learned to control autonomic functions such as blood pressure, vasoconstriction, and heart rate in response to the tones (Dworkin and Dworkin, 1995). Transferring Dworkin’s findings to the problems of learning in CLIS patients, it can be speculated that patients in CLIS would show learning effects due to classical rather than to operant conditioning (Birbaumer, 2006). Thus a paradigm shift from instrumental-operant learning to classical conditioning seems to be necessary to overcome the inability of patients in CLIS to learn BCI control.

The aim of the present study was to develop a new more suitable learning paradigm which could be used even by patients in CLIS and to test it in a first step with a healthy sample. This paradigm is based on semantic classical conditioning of the cortical responses to true and false statements [hereinafter the terms “cortical reactions (CRs)” and “EEG reactions” are used as synonyms].

The concept of semantic conditioning was first characterized by Razran (1939) and refers to the conditioning of a reaction to the meaning of a word or a sentence. Razran showed semantic conditioning of saliva production to words with positive valence and found evidence for transfer to synonyms, but not to homophonic words. In the same way he demonstrated generalization to sentences with identical contextual statements or even emotional valence, independent of the constituent words of the sentences (Razran, 1949, 1961). Previous studies of cortical correlates of differential semantic classical conditioning have shown an increased amplitude of the evoked brain responses (event-related potentials, ERPs) following the onset of the conditioned stimulus (CS; pseudowords or syllables) predictive of an aversive event (Montoya et al., 1996; Heim and Keil, 2006).

From a neurobiological point of view frequent pairing of words with painful or unpleasant stimuli will coactivate neurons involved in processing of language and pain (respectively negative emotions). This would furthermore result in specific cell assemblies being developed. Once the reaction is conditioned these cell assemblies would also be activated in the absence of the unconditioned stimulus (US) leading to CRs to the CS.

In the present study, we investigated cortical evoked responses (using EEG) during and after the pairing of true and false statements with two unpleasant sounds. Differential conditioning of cortical “yes” and “no” responses was achieved using a BCI procedure in healthy participants by pairing two CSs with two different USs. The development of BCI paradigms for binary (yes/no) communication in LIS and CLIS patients has already been reported (Birbaumer et al., 2000; Neuper et al., 2003; Kübler et al., 2005; Naito et al., 2007); however, in the semantic conditioning approach, classical conditioning was investigated for BCI control for the first time.

The study consisted of two experiments: the first experiment (Exp I) tested the feasibility of semantic conditioning in a healthy sample, whereas the second experiment (Exp II) varied features of the USs in two smaller samples and provided a control group without semantic conditioning.

In Exp I we expected (i) conditioned CRs to true and false statements that would be differentiable in the time domain and (ii) classification above chance level for the conditioned ERP reactions to covert “yes” and “no” responses that would allow for basic brain communication (for binary information transfer).

In Exp II, we explored whether different USs (e.g., longer presentation of the USs, autonomous selection of an aversive noise by the participant which then was applied as the US) would improve semantic conditioning. The conditioned cortical “yes” and “no” responses from participants of the conditioning paradigm were also compared to covert “yes” and “no” responses recorded in a paradigm without conditioning. We expect that the cortical responses to affirmative and negative sentences without conditioning cannot be classified on single-trial basis.

The proposed paradigms were developed as such to enable basic affirmative and negative communication and represent a first step toward the restoration of communication by means of BCI in CLIS.

## Materials and Methods

### Participants

Fourteen healthy participants (6 male, 8 female, age range = 21–42, *M* = 24.36, SD ± 5.40) participated in Exp I. All participants were students of the University of Tübingen and took part in the experiment to attain course credits. Exp II included a sample of 18 healthy participants (4 male, 14 female, age range = 19–28, *M* = 22.72, SD ± 2.47) which participated either to attain course credits or for monetary compensation (8€/h). All participants provided informed consent for the study which has been reviewed and approved by the ethical review board of the medical faculty at the University of Tübingen. There was no overlap of subjects between the two experiments. None of the participants reported a history of psychiatric or neurological diseases or showed impaired hearing in a hearing test at the onset.

### Procedure and stimuli

**Figure 1:**
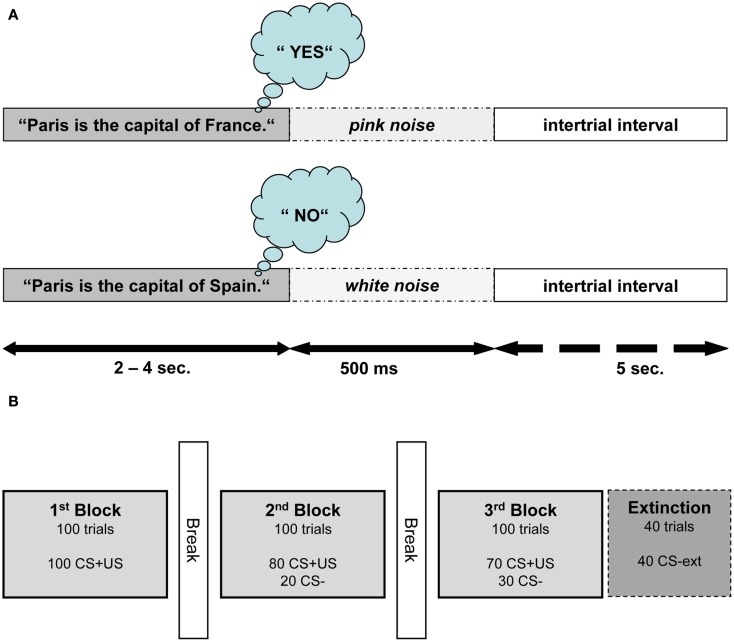
**Structure of semantic conditioning procedure**. **(A)** Depicts the structure of one conditioning trial (CS+US) in Exp I. One trial consisted of a true or false statement followed by 500 ms of noise and an intertrial interval of 5 s. For this example, an English sentence is presented; nevertheless all sentences were presented in German. **(B)** Illustrates the experimental procedure of one session. The extinction block was introduced only in the second session.

All participants attended two conditioning sessions on consecutive days. The participants were seated in a comfortable chair and listened to auditory stimuli presented through pneumatic earphones (E-A-RTONE Gold 3A, Aearo Company, United States). They listened to a total of 640 German sentences (containing an equal amount of true and false statements). The sentences were presented in random order and served as the CSs. The sentences were formulated in a way such that the last word determined if the statement was true or false. Differing only by its last word, each sentence was used both as a true and a false statement (see Figure 1A for an English version of the sentence stimuli used). For the sake of homogeneity of the sentences, the last words did not have more than two syllables. The participants were instructed to wait for the last word and then think “yes” or “no” depending on the content of the statement.

In Exp I, true and false statements (CS1 and CS2) were paired with two USs. True statements were immediately followed by pink noise (US1), presented to the right ear with a duration of 500 ms and an intensity of 75 dB. False statements were followed by white noise (US2) presented to the left ear with duration of 500 ms and an intensity of 105 dB. White noise was used as US because it is perceived as aversive and it was shown to be a successful US in classical conditioning (Neumann and Waters, 2006). Pink noise as US was expected to elicit a different unconditioned reaction (UR) compared to white noise as it corresponds to the frequency range of the human voice and therefore is perceived less aversive. The intertrial interval (ITI) between the end of US and the beginning of the next sentence lasted for 5 s.

Both conditioning sessions consisted of three blocks, with each block containing 100 sentences (50 true and 50 false). Figure 1B illustrates the succession of the blocks within one session. In the first block, every sentence was paired with an US. In the second block 20 sentences (10 true and 10 false randomly selected sentences) were not paired with an US. In the third block, 30 out of 100 sentences were not paired with an US. An additional fourth block (extinction) containing 40 unpaired sentences was presented at the end of the second session. In this last block the resistance of CRs to extinction was examined. Length of the interblock intervals was determined by the participants and had a duration between 1 and 5 min.

In summary, there are three different types of trials (see also Table 1):

- Trials in which each true and false statement is paired with US1, or US2 respectively, to elicit an UR, referred to as CS+US or CS+ trials
- Trials of unpaired true and false statements (CS1−, respectively CS2−) in blocks 2 and 3 which are expected to elicit a conditioned reaction (CR). Every CS− trial is followed by a CS+ trial preventing extinction of the CR.
- Extinction trials: unpaired true and false statements (CS1−ext, CS2−ext respectively) presented at the end of the second session.

**Table 1:** **Different trial types in the conditioning paradigm of Exp I**.

Trial types		No. of trials	Conditioning phase
Paired trials	CS1+US1	250	Acquisition: block 1, 2, 3
	CS2+US2	250	
Unpaired trials	CS1−	50	Intermittent conditioning: block 2, 3
	CS2−	50	
Extinction trials	CS1−ext	20	Extinction: block 4
	CS2−ext	20	

	Total	340	

*The number of trials was calculated over both sessions. CS, conditioned stimulus; US unconditioned stimulus*.

The participants rated the perceived aversiveness of both the US1 and US2 by using the Self-Assessment Manikin (SAM, Bradley and Lang, 1994) at three time points: before the first session (“Pre”) when the USs were presented to the participants for rating, before the start of the conditioning paradigm and at the end of both sessions (“Post”). The SAM consisted of two scales (valence and arousal) for each US, rated on a nine-point Likert scale (1 = positive valence, 9 = negative valence; 1 = high arousal, 9 = low arousal). The participants rated both USs by ticking one of nine boxes below the five depicted manikins for each scale. We expected the arousal and valence of both the USs to be different in order to lead to differentiable CRs. White noise (US2) is expected to be perceived as more arousing and having a more negative valence due to its higher volume and frequency distribution.

In Exp II the participants were divided in three groups each containing six participants. Table 2 presents an overview of the paradigms from both Exp I and Exp II. The paradigm used in Group 1 differs from the paradigm used in Exp I in terms of the length of the US1 and US2. The stimulus duration of pink and white noise was 1000 ms (instead of 500 ms). Group 2 performed the same experiment as the participants in Exp I with the only difference being that US2 was replaced by an individually selected aversive noise (duration of 500 ms and intensity of 105 dB) instead of white noise. Prior to the conditioning, participants listened to seven different aversive sounds (including pink and white noise) taken from a sound battery (Zald and Pardo, 2002). They rated each sound in terms of aversiveness on a visual analog scale (VAS). The sound with the most aversive rating in the VAS scale was used as the US2 and paired with the false statements. Group 3 served as a control group in which no conditioning was applied. These participants listened to the same sentences for two sessions, without the US being presented. They rated the intensity of their “yes” and “no” thinking on a numerical analog scale ranging from 0 (not intense) to 10 (very intense) at the end of both sessions. Participants from Groups 1 and 2 rated the subjective valence and arousal of US1 and US2 on the SAM. The ratings took place at the beginning and end of each session.

**Table 2:** **Description of the paradigms for Exp I and Exp II**.

		CS1	CS2	US1	US2
Exp I		True statements	False statements	Pink noise 500 ms	White noise 500 ms
Exp II	Group 1	True statements	False statements	Pink noise 1000 ms	White noise 1000 ms
	Group 2	True statements	False statements	Pink noise 500 ms	Individually selected noise 500 ms
	Group 3	True statements	False statements	No US	No US

*CS, conditioned stimulus; US, unconditioned stimulus*.

### Data acquisition

The EEG was measured using 32 Ag/AgCl electrodes arranged according a modified version of the 10–10 international system (Oostenveld and Praamstra, 2001) at the positions F3, Fz, F4, T7, C5, C3, C1, Cz, C2, C4, C6, T8, CP5, CP3, CP1, CPz, CP2, CP4, CP6, P5, P3, P1, Pz, P2, P4, P6, PO7, PO3, POz, PO4, PO8, and Oz. The electrodes were referenced to the nose and grounded to the left mastoid and impedances were kept below 5 kΩ. The electrooculogram (EOG) was recorded using two electrodes placed vertically above and below the right eye, and two horizontal EOG channels with the electrodes placed at the outer canthi of the eyes. Data were sampled at 500 Hz and amplified using BrainAmp MR Amplifiers (Brain Products GmbH, Germany) and notch filtered at 50 Hz. Data collection and stimulus presentation were controlled by the BCI2000 (Schalk et al., 2004) and BCPy2000 software.

### Data analysis

#### Preprocessing

After offline-filtering the signal with a high pass filter of 0.009 Hz and a low pass filter of 30 Hz, eye blink artifacts were removed with the Brain Vision Analyzer 2.0 software (Brain Products GmbH, Germany) using independent component analysis (ICA). EEG segments of 2000 ms length were extracted at the end of each statement separately for true and false statements. These segments were offset corrected relative to a 100 ms interval preceding the beginning of the segment.

To investigate if conditioning occurred we first compared the amplitude differences (quantified by using the area under the curve) between the averaged cortical responses of CS+, CS−, and CS−ext trials separately for “yes” and “no” thinking. But as differentiation between averaged cortical responses to affirmative and negative statements is not useful for communication purposes, in a second step we used a radial basis function kernel support vector machine (RBF-SVM) to classify the EEG segments to “yes” and “no” thinking as only successful classification could show the usefulness of this paradigm for communication.

This analysis procedure was carried out for all the experimental paradigms tested.

#### Statistical analysis

##### EEG data

As the visual inspection of the averaged EEG segments of CS1− and CS2− showed no evidence for clear peaked evoked responses, the baseline-corrected area under the curve with respect to increase (AUC_ri_) was calculated for the extracted segments of 2000 ms length. The measure of AUC represents the integral under the EEG curve in a defined time interval. AUC_ri_ reflects the changes over time by ignoring the distance of each data point from zero and instead calculating the integral with reference to the first value of the interval (Pruessner et al., 2003). A time interval of 2000 ms was chosen according to the results of Furdea et al. (2012). To adjust for the different numbers of segments from all the trial types (CS+, CS−, CS−ext), AUC was averaged over the last 20 segments of every trial type, as this is the number of segments available for all of the trial types. The AUC values from all the groups were tested for Gaussian distribution (Shapiro–Wilk). One participant (E1.13) from Exp I had to be excluded from the statistical analysis for the sake of Gaussian distribution. If the assumption of sphericity was violated, Greenhouse–Geisser correction was applied.

The statistical analysis of AUC was carried out to investigate whether conditioning of “yes” and “no” answers was successful and whether the effect of conditioning was maintained even after removal of the US1 and US2 (in CS− trials) and during the extinction phase (in CS−ext trials). For Exp I, repeated-measures ANOVAs were conducted to compare the AUC values from segments corresponding to “yes” thinking (CS1) with AUC values from segments corresponding to “no” thinking (CS2) and with AUC values from the baseline segments. The baseline segments had the same length (2000 ms) and were extracted from the time interval preceding the onset of the sentences. The within-subject factors were: *CS Type* (CS1, CS2, baseline) × *Electrode* (Cz, Pz) × (conditioning) *Phase* (CS+, CS−, CS−ext). Contrasts were calculated for *post hoc* comparisons of within-subject factors.

Identical repeated-measures ANOVAs were conducted for Groups 1 and 2 of Exp II. For Group 3 a repeated-measures ANOVA with the within-factors *Response Type* (CS1−, CS2−, baseline) × *Electrode* (Cz, Pz) was carried out. We expected the CRs to differ between the factor levels of *CS Type*. Furthermore, we anticipated effects of conditioning: the AUC values of the different trial types (levels of the factor *Phase)* were expected to remain similar after removal of the US.

Finally, to evaluate the impact of classical conditioning on the differences in CRs for “yes” and “no” thinking between the conditioning paradigms and the paradigm without conditioning (Exp II Group 3), two repeated-measures ANOVAs were performed. The first ANOVA compared the groups of Exp II, having the within-subject factors *CS Type*, respectively *Response Type* (CS1, CS2, baseline) × *Electrode* (Cz, Pz) and the between-subject factor *Group* (Exp II Group 1, Group 2, and Group 3). In the second ANOVA Exp I was compared with Group 3 of Exp II. The ANOVA included the same within-subject factors and two factor levels (Exp I and Exp II Group 3) for the between-subject factor *Group*.

##### Valence and arousal of the USs

For ease of interpretation the SAM data were recoded for the scale *arousal*, so that after recoding the highest value (9 on a scale from 1 to 9) indicated high arousal. Repeated-measures ANOVAs were performed for the data from Exp I to examine if the subjective rating of valence and arousal for the two USs differed significantly and if they changed over time (within session and between sessions). The within-subject factors were *US Type (*pink noise, white noise) × *Time* (Session1_Pre, Session1_Post, Session2_Post).

For Groups 1 and 2 of Exp II separate repeated-measure ANOVAs were calculated with the same within-subject factors as in Exp I: *US Type* (US1, US2) × *Time* (Session1_Pre, Session1_Post, Session2_Pre, Session2_Post). To compare the ratings of different sounds in Group 2 during selection of the most aversive sound a non-parametric test for repeated measurements (Friedman test) was performed. Changes in the VAS scale ratings of Group 3 between both sessions were analyzed using a paired *t*-test.

Non-parametric tests were chosen for statistical analysis if the requirements for parametric tests were not met. Statistical analysis was performed using SPSS 19. The level of significance was set to α = 0.05.

#### Classification

A radial basis function kernel support vector machine (RBF-SVM) was used for offline classification of the EEG signal. This method showed the best classification accuracies for semantic classical conditioning in a previous study of our group (Furdea et al., 2012). A subset of ten EEG channels (Fz, Cz, CPz, P3, Pz, P4, PO7, POz, PO8, and Oz) was used for classification as these channels provided the best performance and the signal amplitude served as the feature for the classifier. The selection of the 10 electrodes was based on the results of earlier studies that investigated the cognitive processing in different populations (Kotchoubey and Lang, 2003; Krusienski et al., 2006; Nijboer et al., 2008a). These channels were then used for feature extraction. Furthermore, the CR was expected to manifest itself within the first 2 s after the end of a sentence, therefore segments with a length of 2000 ms were extracted from the end of each sentence (first 2000 ms of the ITI). The data segments were filtered using a moving average filter and decimated by a factor of 10. For each trial (sentence) the segments were then concatenated by channels and resulted in a feature vector that was later used to train the classifier. The length of the resulting vector was 1000 samples (1000/10 samples × 10 channels). Single-trial classification results were obtained by training and testing the classifier within a 10-fold cross-validation. During the cross-validation procedure the classifiers could freely choose features from any of the 10 provided channels. The feature selection process was consistent for each participant, but we did not check whether in every given cross-validation step the selected channels were always the same. For details of the classification approach (see Furdea et al., 2012).

The results of this previous study yielded no significant differences in classification accuracy when combining CS− and CS−ext trials for training and testing the classifier compared to using only CS− trials. For this reason, in the classification schemes used here, CS− and CS−ext trials were always combined. To test the applicability of the paradigm for BCI communication, classification of the CRs after unpaired sentences (EEG reactions after CS1− and CS2−, CS1−ext and CS2−ext respectively) is of the most interest. EEG reactions following the sentences paired with an US serve the function of learning the CR and cannot prove the usefulness of the paradigm for BCI communication.

In a first step, classification was applied to separate segments of 2000 ms length after true and false statements which were not paired with the US (CS1− and CS1−ext versus CS2− and CS2−ext), that is, to distinguish between two CRs. Thus, this classification scheme (further referred to as Scheme I) classified segments after true statements (corresponding to “yes” thinking) versus segments after false statements (corresponding to “no” thinking, with 70 trials per class). In a second step, an RBF-SVM was used to classify correctly one of the CR types in comparison to a baseline. For this reason, segments of the same length (2000 ms) preceding the onset of a sentence were extracted as the baseline. In Scheme II, segments following CS1− and CS1−ext (70 trials per class) were classified against the baseline segments. In Scheme III, segments following CS2− and CS2−ext were classified against baseline. For Group 3 of Exp II, 70 CS− trials were selected at random out of the 300 CS− trials per class.

Classification analysis was performed in Matlab R2009b using the LIBSVM toolbox (Chang and Lin, 2001).

## Results

### Statistical analysis

#### EEG data

Only significant main effects and interactions are reported.

The repeated-measures ANOVA for Exp I revealed a significant main effect for *CS Type* [*F*_(2,24)_ = 7.50, *p* < 0.01, partial η^2^ = 0.39]. Contrasts indicated that AUC values corresponding to “no” thinking were significantly higher compared to the AUC values corresponding to “yes” thinking and to the baseline (both *p* ≤ 0.01).

For Group 1 of Exp II the ANOVA yielded significant main effects for *CS Type* [*F*_(2,10)_ = 5.60, *p* < 0.05, partial η^2^ = 0.53]. The contrasts revealed a significantly higher AUC value for segments during “yes” thinking compared to the baseline (*p* < 0.05) and a borderline significance compared to segments of “no” thinking (*p* = 0.059). In Group 2 of Exp II a borderline significant effect of *CS Type* was found [*F*_(1.09, 5.47)_ = 5.82, *p* = 0.055, partial η^2^ = 0.54]. The contrasts revealed a significant difference between AUC values of segments corresponding to “yes” thinking and those corresponding to “no” thinking (*p* ≤ 0.01). For Group 3, in which no conditioning took place, the repeated-measures ANOVA with the within-subject factors *Electrode* and *Response Type* revealed a main effect of the factor *Electrode* [*F*_(1,5)_ = 14.16, *p* < 0.05, partial η^2^ = 0.74] with higher AUC values on Cz.

Statistical comparison of the different groups with the additional between-subject factor *Group* did not reveal any significant main effect of *Group*. This was true for both the group comparison within Exp II and the comparison of Exp I with Exp II Group 3.

#### Valence and arousal of the USs

In Exp I the 2 × 3 repeated-measures ANOVA for valence revealed a significant main effect for the factor *US Type* [*F*_(1,12)_ = 50.79, *p* < 0.001, partial η^2^ = 0.81] indicating that the participants rated white noise (mean rating *M* = 6.77, SD ± 0.33) as more negative than pink noise (*M* = 3.00, SD ± 0.29). A trend for the factor *Time* [*F*_(2,24)_ = 3.19, *p* = 0.059, partial η^2^ = 0.21] was also found. Likewise, the repeated-measures ANOVA for arousal showed a main effect for *US Type* [*F*_(1,12)_ = 48.08, *p* < 0.001, partial η^2^ = 0.80] indicating that white noise (*M* = 5.08, SD ± 1.47) was perceived as more arousing than pink noise (*M* = 3.15, SD ± 1.26). A main effect for *Time* [*F*_(2,24)_ = 8.52, *p* < 0.01, partial η^2^ = 0.42] was found with contrasts revealing significantly lower ratings of arousal after session 2 (S2_Post), compared to session 1 (S1_Pre, S1_Post, *p* < 0.05) for both of the USs.

**Figure 2:**
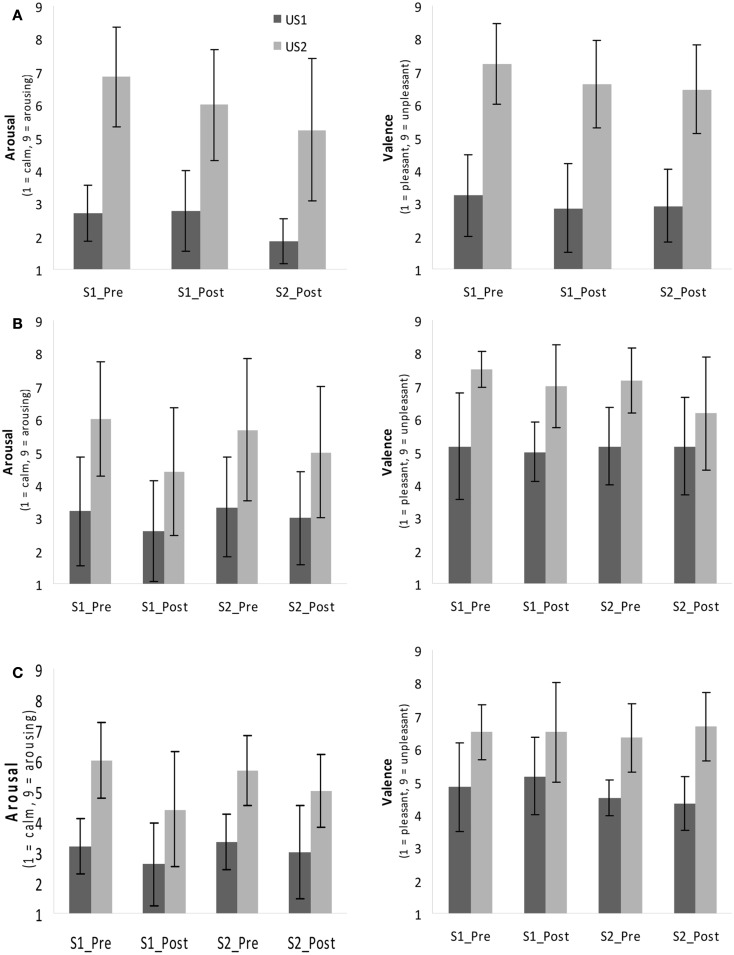
**Rating of valence and arousal (Self-Assessment Manikin, SAM) in Exp I (A) and Exp II for Group 1 (B) and Group 2 (C) before (“Pre”) and after (“Post”) both sessions (S1, S2)**. S1, session 1, S2, session 2.

Figure 2A depicts the ratings of both of the USs and the changes over time for Exp I.

The perceived valence in Group 1 of Exp II differed significantly between US1 (*M* = 4.44, SD ± 0.43) and US2 (*M* = 6.50, SD ± 0.74) as indicated by a main effect of factor *US Type* [*F*_(1,5)_ = 22.00, *p* < 0.01, partial η^2^ = 0.82], but the ANOVA revealed no main effect of the factor *Time*. The ANOVA for arousal showed similar results: a main effect of *US Type* [*F*_(1,3)_ = 10.35, *p* < 0.05, partial η^2^ = 0.78] indicated that the participants rated US2 (white noise, *M* = 4.56, SD ± 1.63) as significantly more arousing than US1 (pink noise, *M* = 2.38, SD ± 0.85).

In Group 2 the repeated-measures ANOVA for valence revealed a significant main effect for the factor *US Type* indicating that the subjects perceived US2 (individually selected noise, *M* = 6.60, SD ± 0.74) as more negative [*F*_(1,5)_ = 38.52, *p* ≤ 0.01, partial η^2^ = 0.77] than US1 (*M* = 4.45, SD ± 0.45). The ANOVA of arousal indicated main effects of both the *US Type* [*F*_(1,4)_ = 54.49, *p* < 0.01, partial η^2^ = 0.93] and *Time* [*F*_(3,12)_ = 4.73, *p* < 0.05, partial η^2^ = 0.54]. Contrasts showed a significantly higher arousal rating before session 1 (S1_Pre) compared to the rating after session 2 (S2_Post, *p* < 0.05). US2 was perceived as significantly more arousing (*M* = 4.40, SD ± 1.05) than US1 (*M* = 2.50, SD ± 0.71). In Figures 2B,C the mean and standard deviation of all ratings in Exp II are depicted; Table 3 lists the mean ratings of all groups.

**Table 3:** **Mean ratings of valence and arousal for US1 and US2 with the SAM**.

	US1		US2	
	Valence	Arousal	Valence	Arousal
Exp I	3.00 (±0.29)	3.15 (±1.26)	6.77 (±0.33)	5.08 (±1.47)
Exp II Group 1	4.44 (±0.43)	2.38 (±0.85)	6.50 (±0.74)	4.56 (±1.63)
Exp II Group 2	4.45 (±0.45)	2.50 (±0.71)	6.60 (±0.74)	4.40 (±1.05)

*Ratings for valence and arousal ranged (after recoding) from 1 (“very pleasant” respectively “not aroused”) to 9 (“very unpleasant” respectively “very aroused”). Standard deviations in parentheses*.

For the selection of the most aversive sound in Group 2 on a VAS scale from 0 to 10, pink noise was rated 3.93 (SD ± 1.52, range 1.3–5.9) and white noise 5.68 (SD ± 0.84, range 4.8–7.2). The most aversive sound which was then used as the US2 in the conditioning was rated as 9.32 (SD ± 0.56, range 8.6–9.9). Four of the participants from Group 2 selected the sound of a train brake as the most aversive, one participant selected the sound of a screeching balloon and the remaining participant the sound of styrofoam squeaking.

A Friedman’s test revealed significant differences (*X*^2^ = 12.00, *p* < 0.01) in the ratings on the VAS scale for white noise, pink noise (used as US1) and the most aversive noise (used as US2). Post hoc tests using a Wilcoxon test found significant differences between white and pink noise (*Z* = −2.20, *p* < 0.05), between white noise and the most aversive sound (*Z* = −2.20, *p* < 0.05) and between pink noise and the most aversive sound (*Z* = −2.20, *p* < 0.05).

Group 3 rated the intensity of “yes” and “no” thinking at the end of every session on a VAS scale from 1 to 10 (1 = not intense, 10 = very intense) with a mean of 7.58 (SD ± 1.24) for session 1 and *M* = 8.5 (SD ± 0.63) for session 2. The ratings did not differ significantly between the sessions [*t*_(5)_ = −2.10, *p* > 0.05].

### Classification

Classification accuracy in Exp I ranged from 43.60 to 62.90% for Scheme I for distinguishing “yes” thinking segments from “no” thinking segments in trials in which the sentences were not paired with the US. Classification of “yes” versus a baseline segment (Scheme II) led to accuracies ranging between 50.00 and 80.70%, whereas classification of “no” versus baseline reached accuracies of 49.30–82.90% (with a chance level of 50% and a confidence interval from 39.00 to 61.00% according to Müller-Putz et al., 2008).

For Group 1 of Exp II, in which longer USs were used for conditioning, classification accuracies ranged between 44.28 and 54.28% for classifying “yes” versus “no” thinking in segments after the end of true and false statement respectively. Classifying “yes” thinking versus a baseline led to accuracies between 41.43 and 77.14% and “no” thinking versus baseline yielded 45.71–76.43% accuracy.

Group 2, which employed an individually selected US2 reached offline classification accuracies between 47.86 and 62.14% for classifying “yes” versus “no” and higher accuracies of 45.00–75.00% for differentiating “yes” versus a baseline segment and 52.10–75.00% for “no” versus baseline.

For the participants of Group 3 the sentences were never paired with any US; the classifier reached classification accuracies between 43.57 and 51.43% for classification of “yes” versus “no”. Classifying “yes” versus a baseline led to 47.86–62.86% accuracy and “no” versus baseline resulted in 51.43–67.14% accuracy.

**Figure 3:**
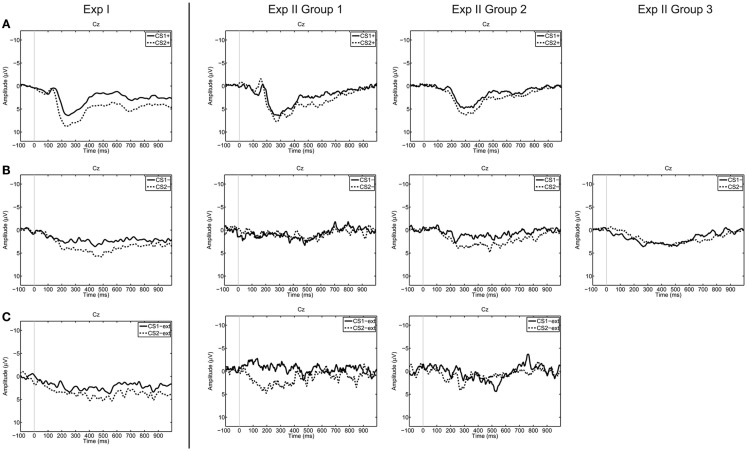
**Grand averages on electrode Cz over all participants, separated for trial types [(A) CS+US trials, (B) CS− trials, (C) CS−ext trials)**. Dashed lines represent trials corresponding to “no” thinking (CS2), the solid lines represent trials corresponding to “yes” thinking (CS1). The grand average was calculated over all available trials for each trial type. From left to right Exp I, Exp II Group 1, Group 2, and Group 3.

The accuracy values of all participants can be found in Table 4, and grand averaged EEG curves separated according to trial types are depicted in Figure 3.

**Table 4:** **Single-trial classification accuracies achieved with RBF-SVM (in %)**.

	Participant	Scheme I	Scheme II	Scheme III
		“Yes” versus “No”	“Yes” versus baseline	“No” versus baseline
**Exp I**				
	E1.1	62.90	56.40	60.70
	E1.2	50.00	50.00	66.40
	E1.3	48.60	59.30	63.60
	E1.4	44.30	76.40	80.00
	E1.5	59.30	70.00	70.00
	E1.6	46.40	50.00	60.70
	E1.7	50.00	80.70	73.60
	E1.8	54.30	68.60	74.30
	E1.9	45.70	71.40	82.90
	E1.10	43.60	74.30	75.70
	E1.11	49.30	67.10	64.30
	E1.12	52.10	72.90	77.90
	E1.13	45.00	60.00	64.30
	E1.14	55.70	58.60	49.30
	**Average**	**50.50**	**65.40**	**68.80**
**Exp II**				
Group 1	G1.1	44.28	77.14	76.43
	G1.2	51.42	59.29	61.43
	G1.3	52.85	67.14	65.00
	G1.4	54.28	41.43	45.71
	G1.5	45.71	47.14	53.57
	G1.6	51.42	61.43	65.71
	**Average**	**49.99**	**58.93**	**61.31**
Group 2	G2.1	54.29	55.71	64.29
	G2.2	51.43	75.00	75.00
	G2.3	62.14	52.86	63.57
	G2.4	51.43	53.57	57.86
	G2.5	50.00	54.29	63.57
	G2.6	47.86	45.00	52.14
	**Average**	**52.86**	**56.07**	**62.74**
Group 3	G3.1	48.57	66.43	54.29
	G3.2	46.43	69.29	67.14
	G3.3	50.71	47.86	51.43
	G3.4	50.71	47.86	55.71
	G3.5	43.57	61.43	65.00
	G3.6	51.43	51.43	55.00
	**Average**	**48.57**	**57.38**	**58.10**

A repeated-measures ANOVA for Exp II was performed to compare the paradigms in terms of classification accuracy for the between-subject factor *Group* (ExpII_G1, ExpII_G2, ExpII_G3) and the within-subject factor *Scheme* (Scheme I, Scheme II, Scheme III). The results indicated a significant main effect of *Scheme* [*F*_(1.20,17.98)_ = 10.03, *p* < 0.01, partial η^2^ = 0.40]. The contrasts revealed that all three schemes significantly differ in accuracy with Scheme III having the highest and Scheme I having lowest accuracy (*p* < 0.05). No main effect of the factor *Group* was found.

A repeated-measures ANOVA compared the results of classification for Exp I and Group 3 of Exp II [*Scheme*(3) × *Group*(2)] and revealed a main effect of both factors. The classification accuracy in Exp I was found to be significantly higher than in Group 3 [*F*_(1,17)_ = 8.09, *p* < 0.05, partial η^2^ = 0.48], which served as the control group without conditioning. For a detailed analysis of the main effect of factor *Scheme* [*F*_(1.51,25.73)_ = 15.68, *p* < 0.001, partial η^2^ = 0.32], the contrasts indicated higher accuracies for Scheme II and Scheme III compared to Scheme I (*p* ≤ 0.001).

## Discussion

The aim of the present study was to develop a new paradigm for binary BCI communication which is based on semantic classical conditioning and requires less controlled attention than traditional BCIs. For the first time two different CRs were conditioned simultaneously using two CSs which differed only according to the validity of a statement.

The usefulness of semantic classical conditioning was investigated in healthy participants and the effects of both semantic conditioning itself and variation of the US were investigated with statistical analysis using AUC_ri_ and single-trial classification with a RBF-SVM.

The results of the statistical analysis of AUC values showed significant effects of *CS Type* (segments corresponding to “yes” significantly differ from those corresponding to “no” thinking) in all conditioning paradigms (Exp I, Exp II Group 1, and Group 2). This indicates that auditory semantic conditioning resulted in two differential CRs for “yes” and “no” thinking. Neither a main effect nor interaction with the factor *Phase* was found, meaning that the difference in CRs between “yes” and “no” thinking was present in all phases of conditioning (acquisition, trials without US during intermittent conditioning, and extinction) and thus, in all trial types (CS+, CS−, CS−ext). In other words, a CR was found in the absence of the US which resembles the URin terms of the AUC and which also did not diminish during the extinction phase.

In the sample in which no conditioning was applied (Group 3), no effect of *Response Type* could be found. This means that segments corresponding to “yes” thinking did not differ from those corresponding to “no” thinking in the AUC values. This clearly indicates the necessity of conditioning in order to be able to differentiate between the EEG reactions related to affirmative and negative sentences.

Even though the effect of *CS Type* (respectively *Response Type*) was found for all groups except for Group 3, the ANOVAs revealed no significant differences between the different conditioning groups and the Group 3 in the AUC measures. Due to the sample size of Exp II statistical power might have been too low to achieve significant group differences for the comparison between the different groups of Exp II. As the direct comparison between the groups lacks evidence we only found indirect indications for the effect of conditioning in the statistical analysis of AUC values as for the experimental group differences between segments corresponding to “yes” and segments corresponding to “no” sentences were significant. Both length and sound of the USs were different between Group 1 and Group 2 of Exp II and therefore confounded the comparison between both groups, but this confound could not influence the assumed differences between the CRs of Group 1 (respectively Group 2) and Group 3.

In all paradigms the US1 (pink noise) and US2 (white noise, individually selected tones respectively) were rated as being different in both the arousal and valence levels. For Exp I (pink noise and white noise with a 500 ms duration) and Group 2 of Exp II (pink noise and individually selected tones with a 500 ms duration) there was a decrease in the perceived arousal reported between the first and the last rating.

The RBF-SVM classified segments after affirmative (“yes” thinking) versus negative (“no” thinking) statements on a single-trial basis with classification accuracies of around 50%. These results on chance level (50 ± 11%) indicate that the classifier was not able to discriminate between two similar ERP reactions occurring in the same time window. As depicted in Figure 3, the morphology of the CRs resembles each other in amplitude and latencies.

In the classification Schemes II and III the CRs (segments corresponding to “yes” for Scheme II, and “no” thinking for Scheme III respectively) were classified against a baseline segment resulting in significantly higher accuracies compared to Scheme I. Accuracies of up to 82.9% were achieved in these classification schemes with mean accuracies of 56.4–68.8% in the different conditioning paradigms (when classifying only one CR against a baseline segment, in Schemes II and III). The highest mean accuracies for all classification schemes were found in Exp I.

The results of the statistical analysis using AUC values of CR and SVM classification of CR differ in terms of the information they reveal. Whilst analysis by ANOVA for the AUC values provided a measurement of differences within a group of participants, the classifier is trained and tested separately for each participant.

Statistical analysis of ERP data tend to underestimate existing differences between two ERP reactions as the averaged responses are sensitive for latency jitters and statistics are biased toward the null hypothesis (Kotchoubey et al., 2002).

In this paradigm the latency jitter might be particularly strong due to differences in the length of the final words which were essential for defining whether a statement was true or false. This could not only influence the averaged ERP response but also account for large variances between individual trials and thus could also be responsible for low classification accuracy. Attempts were made to standardize the length of the last word as much as possible by using only words containing one to two syllables, but the intention was to focus on generating easy and well-understandable sentences.

Investigation of the ERPs following true and false statements without conditioning (in Group 3 of Exp II) should show whether an N400 could be classified on a single-trial basis and used without any conditioning at all for BCI control (Kutas and Hillyard, 1980). A separate investigation of the N400 effect (segmentation related to the beginning of the last word before classification) did not reveal classification accuracies above chance level (50%). This was investigated for both Group 3 of Exp II as well as for all other experimental groups without achieving mean accuracies above 50%.

Based on the results of the AUC values and single-trial classification we conclude that the USs of Exp I (500 ms of pink and white noise) worked best as they achieved the highest classification accuracy, showed significant differences between both the CSs in terms of the AUC values and its classification accuracy differed significantly from the control group (Group 3). The comparison of different groups of Exp II lacks statistical power due to the small sample size, so we cannot interpret the non-significant results of the analysis of group differences within Exp II for both AUC values and classification accuracies.

Nevertheless, the results indicate that semantic conditioning in Exp I was superior to the group without conditioning (Group 3 in Exp II) in terms of classification accuracies, especially when classifying only one CR.

Still the results of Exp II showed that even though the USs in the conditioning groups of Exp II were perceived as aversive and significant different in arousal and valence the variation of the USs did not lead to considerably higher classification accuracies.

In summary, the statistical analysis of the AUC revealed the general efficiency of the semantic conditioning paradigm. The classification of single CRs in Exp I demonstrated the possibility to apply binary communication via BCI, even though the classifier could not differentiate between the two different CRs from one another.

A classification accuracy of 70% has been defined as the criterion level for free BCI communication (Kübler et al., 2001; Choularton and Dale, 2004). In the paradigm presented here, relying on single-trial ERP classification, this criterion has not yet been met. The most commonly applied ERP-based BCI, using averaged P300 responses, has shown higher classification results (Sellers and Donchin, 2006; Nijboer et al., 2008a; Kleih et al., 2010; Mugler et al., 2010), but in all of the cited studies there is a need to average over multiple trials in order to achieve these high accuracies.

Auditory BCIs were only recently developed as a response to numerous reports of impaired eye movement and slowing saccades in ALS patients (Averbuch-Heller et al., 1998; Murguialday et al., 2011). Those BCI systems can achieve mean classification accuracies in healthy participants of around 65–79% using auditory evoked potentials (Furdea et al., 2009; Halder et al., 2010; Schreuder et al., 2010; Höhne et al., 2011), 64% in using auditory feedback for movement imagination (Nijboer et al., 2008b), and 61% in classifying imagined accents in rhythmical patterns for BCI control (Vlek et al., 2011). In a new auditory P300 study with a dynamic stopping method healthy subjects reached mean accuracies up to 86% (Schreuder et al., 2011). Mean accuracies of 68.8% were reached in the semantic conditioning paradigm in Exp I for classifying only the CR after negative statements (corresponding to “no” thinking) against a baseline. However, careful interpretation is required when comparing the classification accuracy results of the current paradigm with classification accuracy results from non-binary BCI paradigms. Taking in account only the binary auditory BCIs paradigms (Nijboer et al., 2008b; Halder et al., 2010; Vlek et al., 2011) the classification results of semantic conditioning when classifying only one CR (Schemes II and III) are comparable. Although a mean classification accuracy of 70% was not reached in this study, a significant main effect of the factor *Group* revealed a higher classification accuracy reached with conditioning (Exp I) compared to classification without conditioning (Exp II Group 3). This result clearly shows the effect of semantic conditioning to increase single-trial classification accuracy for all three schemes used. A possible explanation for the low classification accuracies in differentiating between CRs after true and false statements (classification Scheme I) directly might lay in the similarity of the USs.

The two USs (pink noise and white noise, individually selected noise, respectively), sharing many common physical characteristics (e.g., multiple frequencies) might not have been different enough even though the participants perceived the USs as significantly different both for arousal and aversiveness. The decision to use two auditory USs for differential conditioning was based on the principles of homotopic conditioning (Dworkin and Dworkin, 1999). Using these principles and aiming to achieve stronger CRs, the same sensory channel was used for all stimuli (both CS and US). A study comparing different aversive stimuli for classical conditioning showed that to be salient, US must be perceived as unpleasant. Unpleasant sounds and loud tones lead to effect sizes comparable with electrical shocks in differential conditioning paradigms (Neumann and Waters, 2006).

As the RBF-SVM showed best performance on the level of classifying single CR against a baseline (Schemes II and III), simplification of the paradigm by conditioning only one CR (e.g., only false statements will be followed by an aversive US while the true statements will remain unpaired) could be considered for further studies with semantic conditioning.

But this paradigm (with only one CR conditioned), as well as the classification Schemes II and III face another problem: not finding a response to the particular class which was conditioned (or classified versus baseline, e.g., “no” thinking) does not automatically indicate the participant intended to select the alternative class (e.g., “yes” thinking). This would necessitate providing each statement twice in order to enable real communication: the statement would be re-phrased and presented in both the affirmative and negative forms (“I feel well,” “I feel bad”). Here, the classifier would classify the answer against a baseline in two steps. This should be sufficient for basic communication as every “yes”/“no” question can be reformulated as a negative or affirmative statement.

The sentences used in our paradigm were constructed in a way that an automatic “yes” or “no” answer could have been elicited by the last word. We can however, not be absolutely sure if instead of the meaning of yes and no a possible aftereffect of the stimulus’ impact was captured by the classification in Schemes II and III. Future studies should overcome for this limitation by providing each statement as affirmative and negative sentence and control for consistent classification of both CR to these statements.

Even though two sessions were executed, the number of available trials useable for classification is rather low (70 trials per class). In most of the trials, the CS was paired with the US (CS+ trials) to sustain the CRs. These trials could not be included for classification as they could not provide free communication for BCI control and only serve the goal of conditioning. Further studies should reduce the number of CS+ trials. The results of our previous study (Furdea et al., 2012) showed that classification accuracies did not decrease when taking both the CS− and CS−ext trials for analysis. In other words, even in the extinction phase, in which 40 trials without US were presented, there is no change of the CR in comparison to the CS− trials. Similar results were found in the study presented here, in which no significant change in the AUC values between the CS+, CS−, and CS−ext trials was found. Using an intermittent conditioning paradigm after acquisition phase with a higher number of CS− trials and less CS+ trials would provide faster BCI control (as CS+ trials cannot be used for free BCI communication) and may further strengthen the effect of conditioning (Rescorla and Wagner, 1972).

## Conclusion

In this study, for the first time, two different CR based on CRs were conditioned for “yes”/“no” responses using a semantic classical conditioning paradigm.

The basis of this paradigm for enabling basic BCI communication has been investigated. The next steps will be to test the applicability of the paradigm with severely impaired, LIS, and CLIS patients and to examine learning after potential extinction of goal-directed thinking. Besides testing patients in CLIS with the auditory semantic conditioning paradigm, the effect of different aversive stimuli applied will be examined and online classification and feedback will be implemented in the paradigm.

## Conflict of Interest Statement

The authors declare that the research was conducted in the absence of any commercial or financial relationships that could be construed as a potential conflict of interest.
